# Transient Decrease in Ventricular Sensing Secondary to Left Lung Collapse

**DOI:** 10.7759/cureus.88467

**Published:** 2025-07-21

**Authors:** Julian A Chestaro, Rami N Khouzam

**Affiliations:** 1 Medicine, Grand Strand Medical Center, Myrtle Beach, USA; 2 Cardiology, Grand Strand Medical Center, Myrtle Beach, USA

**Keywords:** bronchoalveolar lavage, complete av block, pacemaker complications, pacemaker lead displacement, permanent pacemaker implantation (ppm)

## Abstract

When interrogating a pacemaker, there are multiple elements that can be measured to provide insight into a pacemaker's integrity and function. Changes in any of those elements outside the normal range may indicate complications such as lead malfunction, lead detachment, or even cardiovascular structural changes. We present a unique case of a patient who underwent permanent dual-chamber pacemaker placement following a bradycardia-induced cardiac arrest. On the first day post-procedure, the patient developed a complete whiteout of the left lung due to a mucus plug, leading to low impedance and low ventricular sensing values on pacemaker interrogation. Remarkably, the sensing values normalized following the resolution of the left lung whiteout after a bronchoalveolar lavage (BAL) successfully removed the mucus plug from the left airway.

## Introduction

A standard pacemaker consists of two main components: a pulse generator and leads. The pulse generator houses the battery and electronics, while the leads extend from the generator to the myocardium to deliver depolarizing pulses and sense intrinsic cardiac activity [1]. Lead wires are typically the weakest part of the pacing system [2], despite new-generation leads having sophisticated advances such as improved geometry and steroid-eluting tips to reduce chronic inflammation and maintain high sensing capability [3]. 

Sensing is the process by which a pacemaker detects the timing of cardiac depolarization in the chamber where the lead is located. Typical electrical amplitudes are expected to range from 1.5 to 5 millivolts (mV) for the atrium and from 5 to 25 mV for the ventricles [1]. If a pacemaker senses amplitudes below 5 mV in the ventricular lead due to structural damage, it can result in inappropriate delivery of pacing pulses, a phenomenon known as undersensing. Impedance, a measure of opposition to the flow of electric current through a circuit, is also monitored to evaluate pacemaker integrity and depends on the properties of the tissues between the electrodes [4]. Normal impedance values typically range from 300 to 1,000 ohms. An impedance below 200 ohms suggests a breach in the leads, although trends are often more significant than single readings [5].

Changes in sensing amplitudes and impedance can be affected by a variety of factors, including anatomical alterations within the thoracic cavity. These changes are often iatrogenic, such as complications related to lead attachment to the myocardium [6]. However, alterations caused by intrinsic airway pathologies that lead to intrathoracic anatomical shifts unrelated to pacemaker placement are rare. We present one such case.

## Case presentation

We report on a 61-year-old male patient with a medical history of cocaine and opioid use disorder who was brought in by emergency medical services for agitation and altered mental status. Opioid overdose was suspected, but the patient's condition did not improve despite the administration of intravenous naloxone. As a result, rapid sequence intubation was performed to secure the airway. Initial workup revealed a positive urine drug screen for cocaine and opioids along with mildly elevated troponin levels. The electrocardiogram (ECG) showed left axis deviation, normal sinus rhythm, and a heart rate of 89 beats per minute, consistent with previous ECGs.

The initial working diagnosis was non-ST-segment elevation myocardial infarction, and the patient was started on dual antiplatelet therapy with aspirin and clopidogrel, along with an angiotensin receptor-neprilysin inhibitor and beta-blockers. Cardiac catheterization was deferred after multiple unsuccessful attempts to wean the patient from mechanical ventilation. Following discussions with the patient’s family, a decision was made to continue life support, and the patient underwent tracheostomy and percutaneous endoscopic gastrostomy tube placement.

Two months later, the patient experienced cardiac arrest. After achieving return of spontaneous circulation, the patient was found to have complete atrioventricular block, and a dual-chamber pacemaker was placed with leads positioned in the right atrial appendage and right ventricle. Intraoperative measurements revealed atrial lead sensing of 1.9 mV with an impedance of 490 ohms and ventricular lead sensing of 6.9 mV with an impedance of 760 ohms.

**Figure 1:**
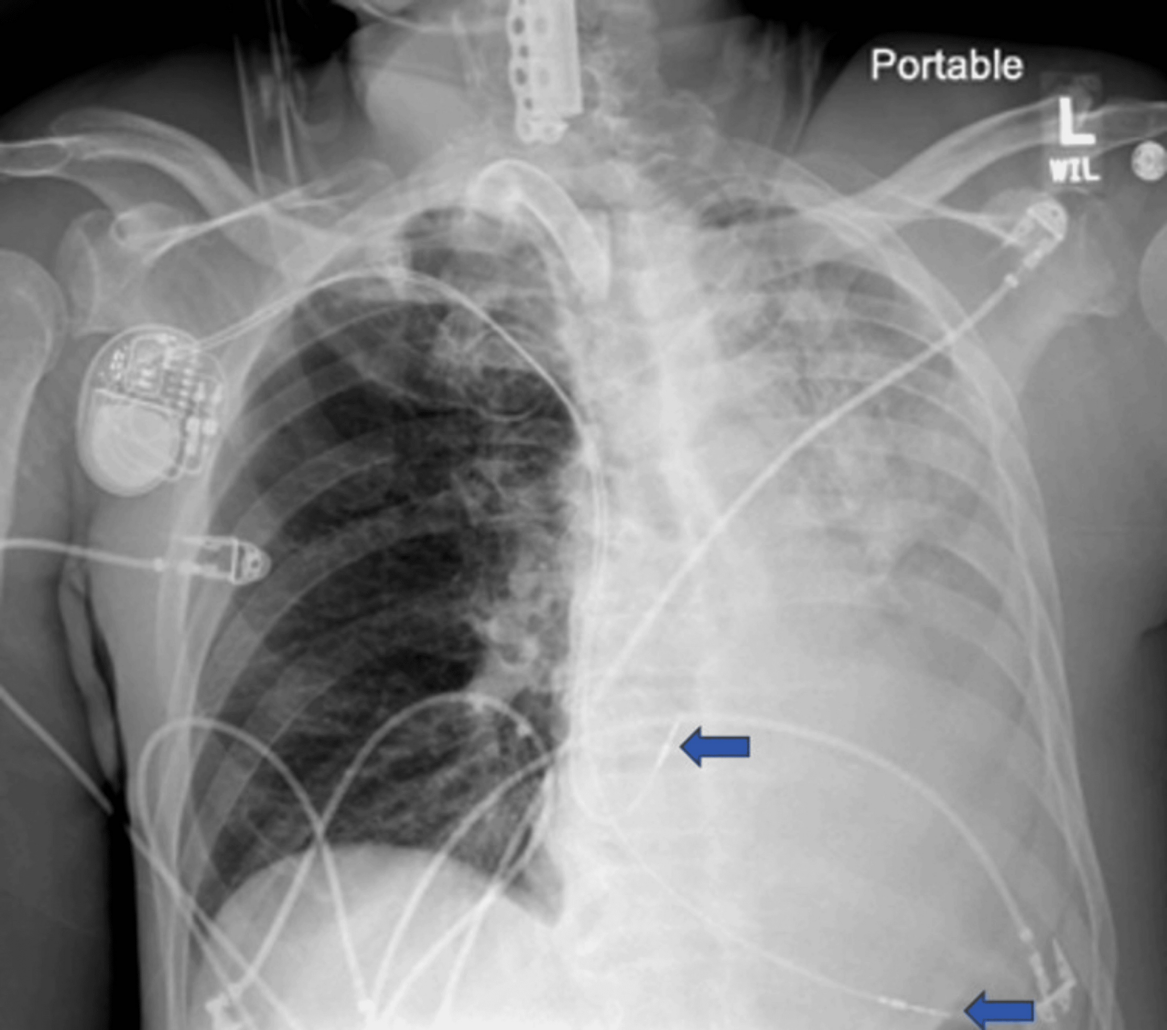
Chest X-ray revealing complete left lung whiteout with a leftward shift of the pacemaker leads (blue arrows).

On the first day post-procedure, the patient developed sudden and severe respiratory distress. A chest X-ray revealed a complete left lung whiteout with a leftward shift of the pacemaker leads (Figure 1).

**Figure 2:**
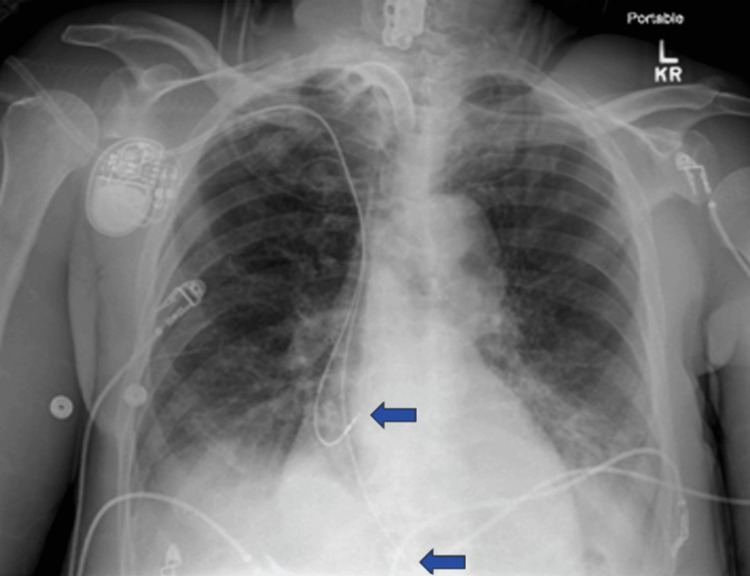
Chest X-ray showing complete resolution of the left-sided whiteout with realignment of the pacemaker leads (blue arrows).

Pacemaker interrogation at that time showed atrial lead readings similar to those obtained intraoperatively, with sensing of 2.1 mV and an impedance of 437 ohms. However, the ventricular lead sensing had decreased to 3.5 mV, and impedance had dropped to 380 ohms, raising concerns about partial lead detachment. An emergent bronchoalveolar lavage (BAL) was performed, successfully removing a mucus plug from the left airway. Following the procedure, the patient’s condition markedly improved. A repeat chest X-ray demonstrated resolution of the left-sided whiteout and realignment of the pacemaker leads (Figure 2).

Pacemaker interrogation post-BAL revealed atrial lead sensing of 4.8 mV, notably higher than before but still within the normal range, with an unchanged impedance of 437 ohms. Remarkably, there was improvement in ventricular lead sensing to 6.6 mV, although the impedance remained low at 361 ohms. A summary of the pacemaker reading during each event can be appreciated below (Table 1).

**Table 1:** Summary of the pacemaker interrogation readings the day the pacemaker was placed, during the left lung collapse, and after BAL. BAL: bronchoalveolar lavage

	Atrial lead		Ventricular lead	
	Impedance (ohms)	P wave (mV)	Impedance (ohms)	R wave (mV)
Pacemaker placement	490	1.9	760	6.9
During lung collapse	437	2.1	380	3.5
After BAL	437	4.8	361	6.6

## Discussion

Pacemakers are essential medical devices. Due to constant mechanical stress from cardiac cycles and bodily movements, physicians must remain vigilant for signs of device integrity compromise during interrogation. In this case, the patient developed a mucus plug in the left airway, resulting in left lung collapse. This led to a leftward shift of the thoracic cardiovascular structures, including displacement of the pacemaker leads. As a result of this shift, ventricular sensing decreased from 6.9 to 3.5 mV, and impedance dropped from 760 to 380 ohms. This case involves an intrinsic airway pathology that led to intrathoracic anatomical shifts, resulting in displacement of the pacemaker leads without detachment. Notably, the changes were reversible and did not require lead revision. To our knowledge, this was an exceedingly rare event, with this case representing the first report to document such an occurrence.

Ventricular sensing values below 5 mV increase the risk of undersensing, which can lead to inappropriate delivery of depolarizing pulses and, subsequently, iatrogenic ventricular arrhythmias [1,7]. The decrease in impedance further supported the diagnosis of partial ventricular lead detachment. Although impedance values below 200 ohms are typically associated with compromised lead integrity and insulation breach, abrupt relative changes in impedance are considered more specific indicators of lead malfunction than absolute measurements [5]. Fortunately, manufacturers are beginning to integrate impedance trend monitoring into device interrogation algorithms to detect early signs of lead compromise, such as Medtronic’s Lead Integrity Alert (LIA) protocol (Minneapolis, Fridley, MN, US) [5,8-10].

An interesting aspect of this case is the near-complete normalization of ventricular sensing following resolution of the lung collapse, as evidenced by post-BAL ventricular sensing levels of 6.6 mV, without the need for ventricular lead revision. Notably, ventricular impedance remained low even after BAL. However, many authors emphasize that while low impedance can suggest lead compromise, interpretation should be made on a case-by-case basis, with greater weight given to trends and serial measurements rather than isolated readings [5].

## Conclusions

The proper functioning of a pacemaker relies on the integrity and correct positioning of its components, particularly the leads, which are susceptible to mechanical stress and failure. In this case, complications such as lead displacement and impaired sensing were identified through imaging and device interrogation, highlighting the importance of careful surveillance of device parameters. Successful intervention with BAL not only improved the patient’s respiratory status but also restored proper lead function without the need for surgical revision. This case underscores the critical role of multidisciplinary management in addressing such complications.
